# Young Women with Early-Stage Breast Cancer Treated with Upfront Surgery: Overview of Oncological Outcomes

**DOI:** 10.3390/jcm13133966

**Published:** 2024-07-06

**Authors:** Lorenzo Scardina, Beatrice Carnassale, Alba Di Leone, Alejandro Martin Sanchez, Ersilia Biondi, Francesca Moschella, Sabatino D’Archi, Antonio Franco, Flavia De Lauretis, Enrico Di Guglielmo, Eleonora Petrazzuolo, Stefano Magno, Riccardo Masetti, Gianluca Franceschini

**Affiliations:** Breast Unit, Department of Women, Children and Public Health Sciences, Fondazione Policlinico Universitario Agostino Gemelli IRCCS, 00168 Rome, Italycarnassale.beatrice@gmail.com (B.C.);

**Keywords:** young women, early breast cancer, breast-conserving surgery, conservative mastectomy, oncological outcomes

## Abstract

**Background**: Breast cancer in young women aged < 40 years is rare and often aggressive with less favorable survival rates. The lack of systematic screening, later stage at diagnosis, and a more aggressive disease biology may all contribute to their poor prognosis. Data on the best management remain conflicting, especially those regarding surgical management, either breast-conserving or mastectomy. To our knowledge, there are limited studies surrounding the treatment of young women with early breast cancer, and this analysis evaluated the oncological outcomes for those patients who underwent surgery upfront. **Methods**: We conducted a retrospective study including 130 young women with early breast cancer from a total of 373 consecutive patients treated with upfront surgery between January 2016 and December 2021 at our institution. Local recurrence-free survival (LR-FS), distant metastasis-free survival (DMFS), disease-free survival (DFS), and overall survival (OS) were evaluated. **Results:** The median follow-up was 61.1 months (range, 25–95). A total of 92 (70.8%) patients underwent breast-conserving surgery, while 38 (29.2%) patients underwent conservative mastectomy with immediate implant breast reconstruction. In total, 8 of 130 patients (6.2%) developed a local recurrence in the treated breast, an7 (5.4%) patients presented distant metastasis. Overall, two (1.6%) patients died due to breast cancer recurrence. **Conclusions**: The results of our study interestingly support breast-conserving surgery in young patients with early-stage breast cancer. While appropriate breast-conserving surgery can achieve favorable oncological outcomes and can always be considered a valid alternative to conservative mastectomy in upfront surgery, a younger age at diagnosis should never be used alone to choose the type of surgery.

## 1. Introduction

Breast cancer is the most frequently diagnosed cancer among women worldwide. Although the incidence of breast cancer is age-dependent, a constant increase in young women has been recently reported [1].

According to the American Cancer Society, about 5% of all breast cancer cases in the United States occur in young women under 40 years of age [2].

The definition of young women in the field of breast oncology is not yet standardized. The European Society for Medical Oncology uses a cut-off of less than 40 years, and accordingly, in this study, we have considered in our analysis patients younger than 40 years [3].

The proportion of young women among all breast cancer patients is not remarkable; however, this is still a prevalent cancer with a less favorable prognosis among young patients [4]. Many studies have shown that worse survival and high risk of recurrence seem to be higher in younger women [5,6]. Actually, breast cancer in young women is characterized by a higher proportion of high-grade, triple-negative, and HER2-positive tumors and has a more aggressive course, less favorable prognosis, and worse survival rates compared to those of older patients.

Breast cancer screening programs in Italy are primarily focused on women aged 50–69 years. Young women are also more likely to present with a self-detected cancer and more likely to be diagnosed at a later stage. Higher breast density in young woman is difficult to interpret on mammography, making diagnostic imaging more challenging [7]. Delayed diagnosis may also be due to a lack of oncological awareness among practitioners who treat young women with breast disease.

Additionally, breast cancer tumors in young women are likely to be larger in size and node-positive and have a higher number of nodes involved than in older women [8].

The more aggressive clinical behavior in younger women compared to older patients is partly due to the tumor immunohistochemical characteristics and distinct subtypes with different prognostic implications, which need to be described. The classification according to four subtypes (Luminal A/B, HER2-positive, and triple-negative) appears to be practical for defining different prognostic subgroups and might be useful in clinical practice.

Hormone receptor-negative HER2-negative (TNBC) or HER2-positive (HER2+) invasive cancers in young women are rare; nevertheless, this cohort presents a higher incidence compared with older women. Moreover, in this particular category of patients, the probability of detecting a mutation is significantly higher than in the general population [9]. The most frequently involved genes are BRCA1/2, and more rarely, TP53. These mutations, particularly BRCA1, are also associated with TNBC [10].

As a result of these considerations, the most aggressive surgical treatment is typically reserved for young women with breast cancer, including those with early-stage breast cancer, which is bilateral mastectomy as opposed to breast-conserving surgery (BCS), despite the absence of inherited breast cancer. Surgery is the most important local treatment for young women, and there is still a lack of studies comparing management and surgical options for oncological outcomes.

Oncoplastic breast surgery involves a shared decision with the patient, wherein surgical treatment is customized to meet the individual needs of each patient. The decision-making process in oncoplastic surgery should aim to balance optimal local disease control with achieving the best reconstructive outcome, always keeping the patient at the center of the process. To achieve this goal, every surgeon involved should be aware of all possible breast surgical and reconstructive options [11,12]. In summary, treatment decisions in young women should not be driven by their age but rather by the biology of breast cancer, to ensure appropriate management and to avoid over-treatment.

Additionally, systemic treatment can be administered before locoregional treatment in this patient category [13]. Neoadjuvant chemotherapy is preferred for young women with TNBC or HER2+ breast cancer, especially when tumor characteristics indicate responsiveness to chemotherapy. [14]. In our sample, we did not consider patients who underwent neoadjuvant chemotherapy, but only considered young women who underwent surgery as their initial treatment.

To date, no comprehensive information or uniform international guidelines exist on the management, treatment, or outcomes of young women with early-stage breast cancer [15,16]. Most of our knowledge about breast cancer is based upon studies in older women, and young women are underrepresented in contemporary research.

Trials dedicated to young patients are essential in addressing many of the unresolved questions and ensuring appropriate treatment.

This retrospective study evaluated the oncological outcomes for those young patients with early-stage breast cancer who underwent surgery upfront.

## 2. Materials and Methods

After obtaining the institutional review board approval of “Fondazione Policlinico Universitario Agostino Gemelli IRCCS—Rome” (ID number: 5766), we retrospectively reviewed the medical records of 373 female patients aged < 40 years diagnosed with invasive breast cancer and treated at our institution between January 2016 and December 2021.

The inclusion criteria were young women aged < 40 years with a primary diagnosis of early invasive breast cancer who received surgery upfront.

Patients with ductal carcinoma in situ, inflammatory breast cancer with evidence of distant metastasis, or those who received neoadjuvant chemotherapy were excluded from the study.

Of 373 women reviewed, 130 patients met the eligibility criteria and were included in the current analysis.

A complete preoperative workup, including clinical assessment, breast imaging exams, and disease staging, was performed for all patients. Surgical planning was discussed in a multidisciplinary meeting. A specific algorithm shared with the plastic surgeons, based on anamnestic, morphological, functional, and oncological criteria, was used to define the most appropriate surgical technique.

Conservative mastectomy with immediate breast reconstruction was performed on patients with extensive or multicentric cancers and a tumor-to-breast volume ratio that required the excision of >50% of the glandular volume, tumors in which clear surgical margins with BCS were not obtained, BRCA mutations, contraindications to adjuvant radiotherapy, and according to patient preference. The reconstruction was carried out with breast implants. Breast-conserving surgery with level II oncoplastic techniques was provided to patients for whom a standard conservative procedure with safe margins would have appeared unfeasible or would have resulted in greater deformity. In all other cases, patients underwent BCS with level I oncoplastic techniques.

The purpose of the present study was to assess the outcomes in terms of local recurrence-free survival (LRFS), distant metastasis-free survival (DMFS), disease-free survival (DFS), and overall survival (OS).

LRFS was calculated as the period from the date of surgical treatment to the date of local recurrence. DMFS was calculated as the period from the date of surgery to the date of detection of distant metastases. DFS was calculated as the period from the date of surgical treatment to the date of any tumor progression, including local recurrence or distant metastases. OS was calculated as the time interval from surgical treatment to death from any cause.

Follow-up was performed every six months. Results are expressed as means with standard deviations and medians with ranges.

Univariate descriptive analysis of the variables under study was carried out by calculating the centrality and variability indices for the quantitative variables and using frequency tables for the qualitative variables. Survival from events was assessed using a Kaplan–Meyer curve, and the curves of different subgroups were compared using the log-rank test. An alpha significance level of 0.05 was used in all analyses mentioned. For the statistical analysis of data, IBM SPSS Statistics software version 28.0 for Windows was used.

## 3. Results

A total of 130 young women with early-stage breast cancer were included. The median follow-up was 61.1 months (range, 25–95). A total of 92 (70.8%) patients underwent BCS, while 38 (29.2%) patients underwent conservative mastectomy with immediate implant breast reconstruction. Regarding axillary surgical treatment, 107 (82.3%) patients underwent sentinel lymph node biopsy (SLNB) and 23 (17.7%) underwent axillary lymph node dissection (ALND) after SLNB. Ductal invasive carcinoma was found in 117 (90%) patients, and 8 (6.1%) had lobular invasive carcinoma, while 5 (3.9%) patients were diagnosed with other histologic types of cancer. Considering tumor grade, 18 (13.8%) were grade 1 (well differentiated), 67 (51.6%) were grade 2 (moderately differentiated), and 45 (34.6%) were grade 3 (poorly differentiated). Luminal tumors were observed in 95 (73.1%) women; 18 (13.8%) were HER2-positive and 17 (13.1%) were TNBC. Germline mutations in BRCA1/2 were present in 17 (13.0%) patients; 15 (11.5%) patients had the BRCA 1 mutation, and 2 (1.5%) patients had the BRCA 2 mutation. Patients’ characteristics and clinicopathological features are summarized in Table 1.

**Figure 1:**
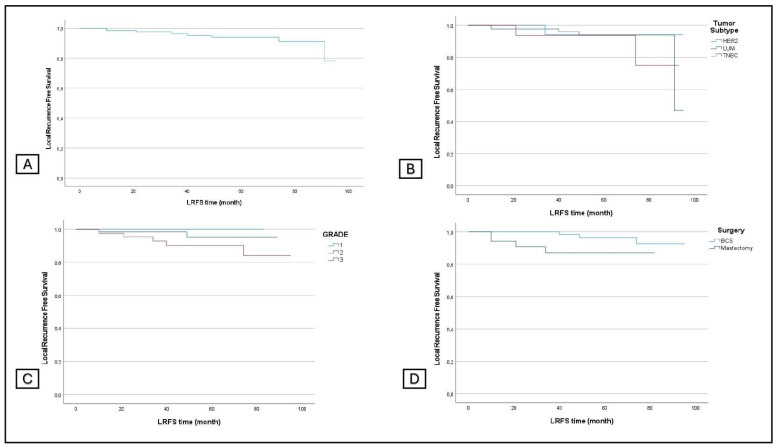
Local recurrence-free survival in all patients (**A**) and in subgroup analyses stratified by tumor subtype (**B**), grading (**C**), and surgery (**D**).

The overall LRFS was 93.8%, and the LRFS by grade was 100% for grade 1, 95.5% for grade 2, and 88.9% for grade 3. Concerning the subtype, LRFS was 94.7% for luminal tumors, 88.2% for TNBC, and 94.4% for HER2+. Regarding the surgical procedures, LRFS was 96.7% in the BCS group and 86.8% in the conservative mastectomy group (*p*: 0.011) (Figure 1).

**Figure 2:**
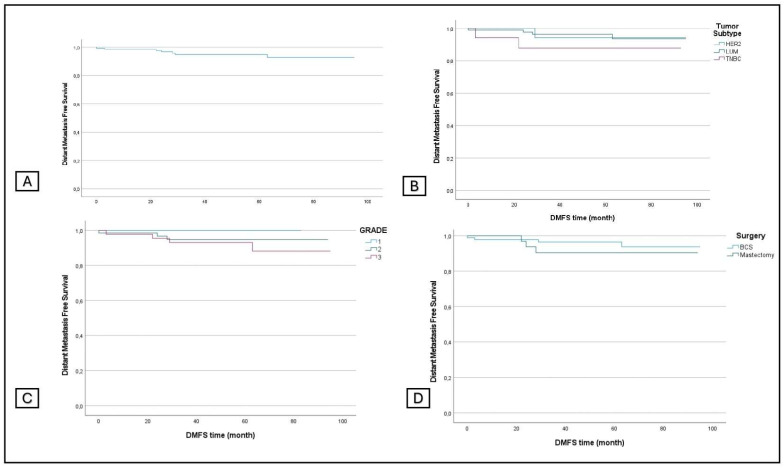
Distant metastasis-free survival in all patients (**A**) and in subgroup analyses stratified by tumor subtype (**B**), grading (**C**), and surgery (**D**).

The overall DMFS was 94.6%, and the DMFS by grade was 100%, 95.5%, and 90.1% for grades 1, 2, and 3, respectively. The DMFS by tumor subtype was 95.8%, 88.2, and 94.4% for LUM, TNBC, and HER2+, and the DMFS by surgical procedure was 95.7% for BCS and 92.1% for conservative mastectomy (Figure 2).

**Figure 3:**
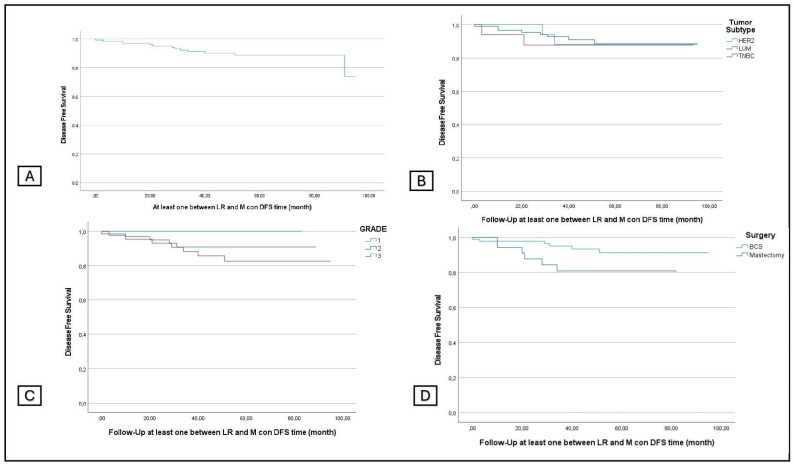
Disease-free survival in all patients (**A**) and in subgroup analyses stratified by tumor subtype (**B**), grading (**C**), and surgery (**D**).

The overall DFS was 90%, and the DFS according to the grading was 100%, 92%, and 84.4% for grades 1, 2, and 3, respectively. The DFS was 90.5%, 88.2%, and 88.9% according to tumor subtype and was 93.5% and 81.6% for BCS and conservative mastectomy (p: 0.026) (Figure 3).

**Figure 4:**
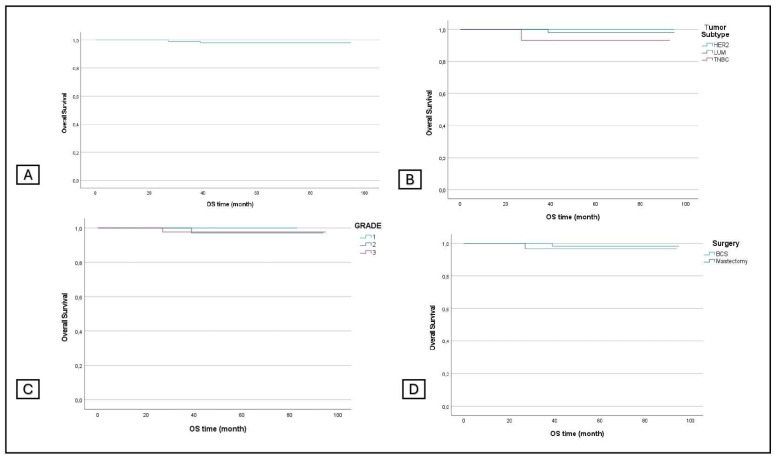
Overall survival in all patients (**A**) and in subgroup analyses stratified by tumor subtype (**B**), grading (**C**), and surgery (**D**).

The OS was 98.5%, and the OS according to the grading was 100%, 98.5%, and 97.8%. The OS according to the subtype was 98.9% for luminal tumors, 94.1% for TNBC, and 100% for HER2+; the OS according to surgical procedures was 98.9% and 97.4% for the two groups, respectively (Figure 4).

Comparisons of oncological outcomes based on grading, type of surgery, and tumor subtype are summarized in Table 2.

## 4. Discussion

In this study, the management, risks, and prognostic factors of all surgically treated young patients with early-stage breast cancer were analyzed. Data on the best surgical treatment for these patients remain conflicting, especially those regarding BCS or mastectomy. There is no evidence that mastectomy improves OS in young breast cancer patients [17], so BCS in association with oncoplastic techniques should be considered [18]. When mastectomy is indicated, skin- and nipple-sparing with immediate breast reconstruction should therefore be offered. Significantly, BCS has shown survival benefits that are at least comparable to those of mastectomy, with even more favorable outcomes observed in young patients [19].

Voogd et al. [20] reported that very young patients who received BCS had a higher local recurrence risk compared to those who received mastectomy. The same findings were reported by Van der Sanger et al. [21], in which the rates of local recurrence in young mastectomy patients (not very young) were lower than those in the breast-conserving surgery group. There were no differences in overall survival rates between the surgical arms. In our study, 92 (70.8%) patients underwent BCS in association with adjuvant radiation therapy, while 38 (29.2%) patients underwent skin- or nipple-sparing mastectomy with immediate implant breast reconstruction. Patients’ characteristics, categorized by the type of surgical intervention, are summarized in Table 3.

During the investigation period, the number of local recurrences in BCS patients was lower than in the mastectomy group with a statistically significant difference (*p* = 0.011). Moreover, DFS was statistically significant (*p* = 0.026). Overall, in patients undergoing BCS, six events (6.5%) occurred, while in the conservative mastectomy group, there were seven (18.4%).

Our findings regarding relapses in the mastectomy group compared to the BCS group suggest that patients who underwent mastectomy were generally affected by multifocal tumors, with a higher risk of recurrence. These results were also supported by adjuvant radiotherapy after BCS, which plays an important role in enhancing local control of recurrence. Following BCS, radiation therapy reduces the rate of disease relapse and decreases the breast cancer mortality rate [22,23].

The EBCTCG conducted a large meta-analysis and found that adjuvant radiation therapy combined with BCS could significantly reduce the rate of local recurrence and mortality over the subsequent 15 years [24]. Furthermore, the higher rate of local relapses in patients undergoing conservative mastectomy did not influence overall survival, and there was no significant difference between the two groups (*p* = 0.472). These data allow us to state that BCS associated with adjuvant radiotherapy, compared to conservative mastectomy, worsens neither the risk of local relapse nor distant recurrence. However, some study limitations must be considered, such as the small number of patients, the average follow-up of 60 months, and the retrospective nature of the analysis.

The results of this analysis regarding overall survival confirm what has already been reported in the literature. De Boniface et al. [25] demonstrated that young patients who underwent BCS had a significantly better 5-year overall survival rate than those who underwent mastectomy. In our study, BCS plus radiotherapy seems to be at least equivalent to conservative mastectomies in young women, and the increasing use of mastectomy in these patients may no longer be justified with the assumption of better survival.

Therefore, age should not be considered the main factor that directs the choice of surgical treatment; several evaluations must be made.

Regarding axillary surgery, SLNB is considered for nodal staging in patients with early breast cancer and clinically negative nodes, while axillary dissection is indicated in breast cancers with clinically positive nodes. In our sample, all patients underwent SLNB, but despite the early stage, approximately 17% of patients underwent ALND after SLNB due to macrometastases on intraoperative frozen section examination, probably because of the aggressive biological nature of certain tumor subtypes. It is mandatory to mention new therapeutic strategies that are also emerging for young women; axillary surgery, and even SLNB, can be omitted entirely for patients with small breast cancers, without any effect in terms of distant disease-free survival at 5 years [26,27].

TNBC and HER2-positive disease are more prevalent in younger patients than in older women [28], and in line with the literature, our study also showed that these patients more often have an aggressive subtype of breast cancer. In this series, young patients with more aggressive subtypes have the same risk of recurrence when compared with luminal tumors. Despite the more aggressive histotype of these tumors, a statistically significant difference in terms of LRFS, DFS, DDFS, and OS was not found in our sample (Table 2). All of this may be because only small-sized tumors were considered, and furthermore, in recent years, the use of increasingly advanced adjuvant therapies has been observed, especially in aggressive subtypes.

Based on our analysis with regard to grading, there are no statistically significant differences in terms of LRFS, DMFS, DFS, and OS. However, we have to highlight that the trend is unfavorable for patients with poorly differentiated breast cancer, particularly concerning LRFS and DFS. Probably, in this case, the absence of statistical significance is related to the lack of long-term follow-up.

Nowadays, all young patients with early-stage breast cancer should undergo genetic preoperative testing, and even in cases of identified pathogenetic variants, the possibility of breast-conserving surgery should still be discussed.

Patients with breast cancer at a young age were more likely affected by the gene pathogenic variants BRCA1/2 than the general population [9], and in our study, we observed seventeen patients with BRCA1/2 mutations (13.0%). Young BRCA mutation carriers showed a poorer prognosis in terms of recurrence and survival compared with non-carriers [29]. The majority of BRCA mutation carriers in our sample underwent genetic testing post-operatively. Genetic counseling should be offered for every young woman, regardless of family history or tumor subtype, and genetic testing should be performed only after obtaining adequate information about the implications of the results [28]. In the current era of personalized medicine, the surgical approach to inherited breast cancer requires more appropriate counseling on different surgical strategies to offer the best care [9,29].

During the study observational period, from January 2016 to December 2021, 179 young patients with breast cancer were excluded from our sample as they underwent neoadjuvant chemotherapy. Young women with breast cancer are more likely than older women to have tumors with aggressive phenotypes, and consequently, they will benefit from neoadjuvant chemotherapy. It may provide important advantages: first of all, downstaging tumors often allows clinicians to increase the proportion of patients who are eligible for BCS [30]. In our sample, we only considered patients with early-stage breast cancer who had not undergone neoadjuvant chemotherapy. It could be interesting to analyze the oncological outcomes by comparing young women with breast cancer who, at the same stage and tumor phenotype, underwent neoadjuvant chemotherapy versus upfront surgery [31,32].

However, breast-conserving-surgery-eligible patients often choose mastectomy (most frequently, bilateral) regardless of their response to neoadjuvant chemotherapy. While a greater awareness of genetic and familial high risk has been proposed as a factor responsible for young women with breast cancer choosing mastectomy, personal preference without a known high-risk predisposition is the most common reason for choosing mastectomy [33].

In conclusion, we believe that young women with early-stage breast cancer represent a distinct category of patients and surgical treatment should be discussed in multidisciplinary meetings, while always considering genetic testing, risk prediction tools, and the best surgical management in accordance with the patient’s wishes [34].

The main limitations of the study were the sample size, the retrospective design, and the single-center analysis. Young women with early breast cancer are a unique group of patients who may need further investigation.

## 5. Conclusions

Breast cancer in young women is becoming more prevalent and a significant clinical issue. Diagnosis and surgical treatment strongly impact the quality of life of these patients. The management of young women with early-stage breast cancer needs a standardized approach involving a multidisciplinary team to ensure specific and optimal treatment. Despite the limitations related to the analysis, these results interestingly support BCS in young patients with early-stage breast cancer. Even if conservative mastectomy has traditionally been considered the first choice for young women, BCS seems to be at least equivalent in term of oncological outcomes in early-stage breast cancer. The increasing use of mastectomy in young patients may no longer be justified on the basis of improved survival. Although there are still no prospective randomized trials comparing different surgical options in young women with early-stage breast cancer, according to these data, we could assume that BCS should always be considered in selected patients. Nevertheless, our study seems to confirm that young women with breast cancer present peculiar oncological and clinical issues that impact the surgical treatment and outcome. There is a strong need to acquire more consistent data on this patient category to better define optimal management and to standardize and spread new strategies that can provide significant benefit to young women with early-stage breast cancer.

## Figures and Tables

**Table 1:** Characteristics and clinicopathological features of 130 young women with early-stage breast cancer.

**Patients**	130
**Age (years)**	36 (25–40)
**Histologic subtype**	
- Ductal invasive carcinoma	117 (90.0%)
- Lobular invasive carcinoma	8 (6.1%)
- Others	5 (3.9%)
**Grading**	
- 1	18 (13.8%)
- 2	67 (51.6%)
- 3	45 (34.6%)
**Stage**	
- pT1mi	6 (4.6%)
- pT1a	6 (4.6%)
- pT1b	33 (25.4%)
- pT1c	85 (65.4%)
- pN0	89 (68.5%)
- pN0 (i+)	4 (3.0%)
- pN1mi	13 (10%)
- pN1	21 (16.2%)
- pN2	3 (2.3%)
- Multifocal	37 (28.5%)
**Biological subtypes**	
- Luminal-like	95 (73.1%)
- HER2-enriched	18 (13.8%)
- Triple-negative	17 (13.1%)
**Adjuvant treatment**	
- Radiotherapy	95 (73.0%)
- Hormone therapy	106 (81.5%)
- Chemotherapy	57 (43.9%)
**Surgical treatment**	
- Breast-conserving surgery	92 (70.8%)
- Conservative mastectomy	38 (29.2%)
- SLNB	107 (82.3%)
- ALND	23 (17.7%)
**BRCA 1**	15 (11.5%)
**BRCA 2**	2 (1.5%)

**Table 2:** Oncological outcomes of 130 patients.

	LRFS *p*-Value (Chi-Square)	DMFS *p*-Value (Chi-Square)	DFS *p*-Value (Chi-Square)	OS *p*-Value (Chi-Square)
**Tumor Subtype**	0.763	0.442	0.981	0.315
	(0.613)	(1.632)	(0.039)	(2.313)
**Grading**	0.454	0.412	0.320	0.842
	(1.581)	(1.774)	(2.276)	(0.345)
**Type of Surgery**	**0.011**	0.370	**0.026**	0.472
	(6.420)	(0.803)	(4.927)	(0.518)

**Table 3:** Characteristics and clinicopathological features of 130 young women with early-stage breast cancer according to the surgical treatment.

	Mastectomy	BCS
**Patients**	38 (29.2%)	92 (70.8%)
**Age (years)**	36.7 (25–40)	36.9 (27–40)
**Histologic subtype**		
- Ductal invasive carcinoma	36 (94.8%)	81 (88%)
- Lobular invasive carcinoma	2 (5.2%)	6 (6.5%)
- Others	0 (%)	5 (5.5%)
**Grading**		
- 1	5 (13.2%)	13 (14.2%)
- 2	19 (50%)	48 (52.2%)
- 3	14 (36.8%)	31 (33.6%)
**Stage**		
- pT1mi	3 (8%)	3 (3.3%)
- pT1a	5 (13.2%)	1 (1%)
- pT1b	7 (18.4%)	26 (28.3%)
- pT1c	23 (60.4%)	62 (67.4%)
- pN0	26 (68.5%)	63 (68.5%)
- pN0 (i+)	0 (%)	4 (4.4%)
- pN1mi	2 (5.2%)	11 (11.9%)
- pN1	10 (26.3%)	11 (11.9%)
- pN2	0 (%)	3 (3.3%)
- Multifocal	18 (47.4%)	19 (20.7%)
**Biological subtypes**		
- Luminal-like	27 (71%)	68 (74%)
- HER2-enriched	4 (10.6%)	14 (15.2%)
- Triple-negative	7 (18.4%)	10 (10.9%)
**Adjuvant treatment**		
- Radiotherapy	7 (18.4%)	88 (95.6%)
- Hormone therapy	26 (68.5%)	80 (87%)
- Chemotherapy	22 (57.9%)	35 (38%)
**Axillary surgery**		
- SLNB	27 (71%)	80 (87%)
- ALND	11 (29%)	12 (13%)
**BRCA 1**	9 (23.7%)	6 (6.5%)
**BRCA 2**	1 (2.6%)	1 (1%)

## Data Availability

The data presented in this study are available on request from the corresponding author.
